# A Huge Plantar Intramuscular Hemangioma in the Plantar Area Treated Surgically: A Case Report and Literature Review

**DOI:** 10.3390/ijerph18179088

**Published:** 2021-08-28

**Authors:** Hong Seop Lee, Yong Cheol Hong, Ki Jin Jung, Eui Dong Yeo, Sung Hun Won, Si-Hyong Jang, Jae Young Ji, Dhong Won Lee, Sung Joon Yoon, Woo Jong Kim

**Affiliations:** 1 Department of Foot and Ankle Surgery, Nowon Eulji Medical Center, Eulji University, Hangeulbiseok-ro, Nowon-gu, Seoul 01830, Korea; sup4036@naver.com; 2 Department of Orthopaedic Surgery, Soonchunhyang University Hospital Cheonan, 31, Suncheonhyang 6-gil, Dongam-gu, Cheonan 31151, Korea; ryanhong90@gmail.com (Y.C.H.); c89546@schmc.ac.kr (K.J.J.); yunsj0103@naver.com (S.J.Y.); 3 Department of Orthopaedic Surgery, Veterans Health Service Medical Center, Seoul 05368, Korea; angel_doctor@naver.com; 4 Department of Orthopaedic Surgery, Soonchunhyang University Hospital Seoul, 59, Daesagwan-ro, Yongsan-gu, Seoul 04401, Korea; orthowon@schmc.ac.kr; 5 Department of Pathology, Soonchunhyang University Hospital Cheonan, 31, Suncheonhyang 6-gil, Dongam-gu, Cheonan 31151, Korea; slogic001@naver.com; 6 Department of Anesthesiology and Pain Medicine, Soonchunhyang University Hospital Cheonan, 31, Suncheonhyang 6-gil, Dongam-gu, Cheonan 31151, Korea; phmjjy@naver.com; 7 Department of Orthopaedic Surgery, Konkuk University Medical Center, 120-1, Neungdong-ro, Gwangjin-gu, Seoul 05030, Korea; bestal@naver.com

**Keywords:** plantar, hemangioma, intramuscular, foot pathology

## Abstract

Intramuscular hemangioma (IH) is rare, accounting for only 0.8% of all hemangioma cases. In particular, IH of the foot has only been reported a few times. In such cases, the symptoms typically include tenderness and swelling, often in relation to physical activity, but tingling or impaired function may also be present. Here, we report a patient who presented with a significant IH in the plantar area treated surgically. A 25-year-old female visited our hospital with pain in the plantar aspect of the right foot. She had noticed a mass about 10 years prior. She had previously experienced pain only when pressing the mass, but the pain subsequently became more regular pain and was exacerbated by exercise. In fact, the pain became so intense that she could not sleep well. Upon physical examination, mild swelling and tenderness of the plantar area were noted in the second to the fourth metatarsal. Sensation and motor reflexes were normal and the results of Tinel’s test were negative. Plain radiographs of the right foot revealed phleboliths scattered throughout the first to third intermetatarsal spaces. Magnetic resonance imaging revealed a space-occupying multilobulated mass (5.6 × 2.8 × 2.5 cm) located in the flexor digitorum brevis (FDB) muscle, which penetrated the plantar fascia and spread to the subcutaneous layer. In T2-weighted images, the lesion displayed a hyperintense signal compared to the surrounding skeletal muscle. Based on radiological findings, we suspected IH. The mass surrounded by the FDB muscle was exposed and completely removed via wide excision. IH consisting of cavernous-like vascular structures was diagnosed on pathology. At 1-year follow-up, the patient was almost asymptomatic and had recovered almost full range of motion in the plantar area. Histological analysis and surgery are recommended to remove intramuscular hemangiomas in the plantar area, but if the patient is not suitable for surgery, sclerotherapy or combination treatment should also be considered.

## 1. Background

Hemangioma is a common benign soft tissue tumor that accounts for 7–10% of all soft tissue tumors [1]. However, intramuscular hemangioma (IH) is rare, accounting for only 0.8% of all hemangiomas [2]. The lower extremities are the most commonly affected sites, particularly the thigh [3,4]. The incidence of IH of the foot has not been reported accurately, and there have been few reports of cases, especially occurring on the plantar, area in the literature [5,6,7,8,9,10]. The symptoms in such cases are mostly tenderness and swelling, which are often related to activity, but tingling or impaired function caused by compression of the surrounding nerve structures may also occur, however, some cases are asymptomatic [2,5,9,11]. Since these symptoms are non-specific, which may appear in other tumors occurring in soft tissue, a diagnostic dilemma may occur. Diagnosis is mainly based on magnetic resonance imaging (MRI), but histologic examination of the excised tumor is necessary for confirmation. For symptomatic IH in the plantar area, surgical resection is the treatment of choice [2,4,12,13]. Surgical excision allows for a definitive diagnosis and reduces the risk of recurrence. However, in some cases, resection may not be possible, so injection of a sclerotic agent may be considered an alternative treatment [8,9]. 

Here, we report a patient who presented with a huge IH in the plantar area that was treated surgically. This case report demonstrates a unique presentation of IH due to its rare localization of the foot.

## 2. Case Presentation

This case report was approved by the Institutional Review Board and Human Research Ethics Committee of Soonchunhyang University Cheonan Hospital (IRB No. 2020–03–004). The patient gave written informed consent for publication of this report and the accompanying images.

### 2.1. Preoperative Evaluation

A 25-year-old female visited our hospital with pain in the plantar aspect of the right foot. About 10 years prior, she had noticed a mass but ignored it, as she only felt pain on pressing the mass. However, over time, the pain became persistent and was also exacerbated by exercise. She could not sleep well due to the pain.

The patient had a normal foot structure and no history of trauma. She did not have rheumatoid arthritis or other systemic diseases, did not take any medication, and did not have a history of steroid injections into the affected foot. The patient visited a local clinic and plain X-ray revealed calcification. Her pain continued to worsen and she was transferred to our hospital for further evaluation.

**Figure 1:**
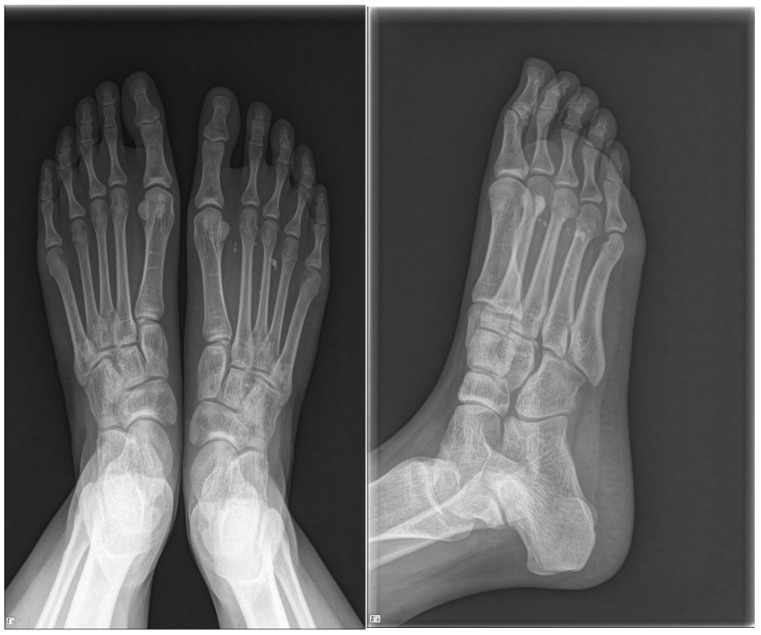
Plain radiographs of the right foot showing phleboliths scattered throughout the first to third intermetatarsal spaces.

**Figure 2:**
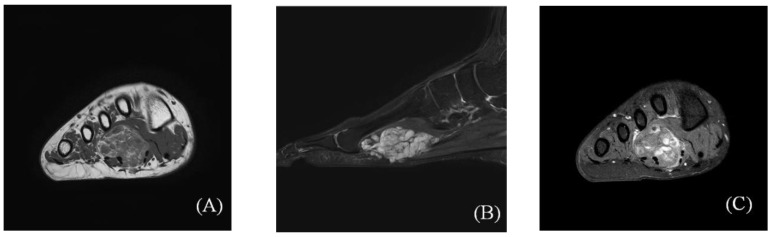
Preoperative magnetic resonance imaging (MRI) scans showing a 5.6 × 2.8 × 2.5 cm multilobulated soft tissue mass in the flexor digitorum brevis muscle, which penetrates the plantar fascia and spreads to the subcutaneous layer. (**A**) Axial T1-weighted images; (**B**) sagittal T2-weighted images; (**C**) axial gadolinium T1-weighted image.

Upon physical examination, mild swelling and tenderness were observed in the plantar area, from about the second to the fourth metatarsal. Though not visible, the mass was palpable. Sensation and motor reflexes were normal and the results of Tinel’s test were negative. Plain radiographs of the right foot revealed phleboliths scattered throughout the first to third intermetatarsal spaces (Figure 1). MRI revealed a space-occupying multilobulated mass (5.6 × 2.8 × 2.5 cm) in the flexor digitorum brevis (FDB) muscle, which penetrated the plantar fascia and spread to the subcutaneous layer (Figure 2). In T1-weighted images, the mass had intermediate signal intensity (Figure 2A). In T2-weighted images, the lesion displayed a hyperintense signal compared to the surrounding skeletal muscle (Figure 2B). The mass showed heterogeneous partial peripheral enhancement in T1-weighted images after gadolinium injection (Figure 2C). Based on these findings, we suspected IH.

The authors explained the sclerotherapy or surgery to the patient, but the patient requested surgery because the severe pain interfered with her daily life and was negative about multiple treatments. The preoperative visual analog scale (VAS) pain score and American Orthopedic Foot and Ankle Society (AOFAS) midfoot scale score were 7 and 87, respectively. We planned elective general surgery and explained the basic procedures to the patient. 

### 2.2. Surgical Procedure

**Figure 3:**
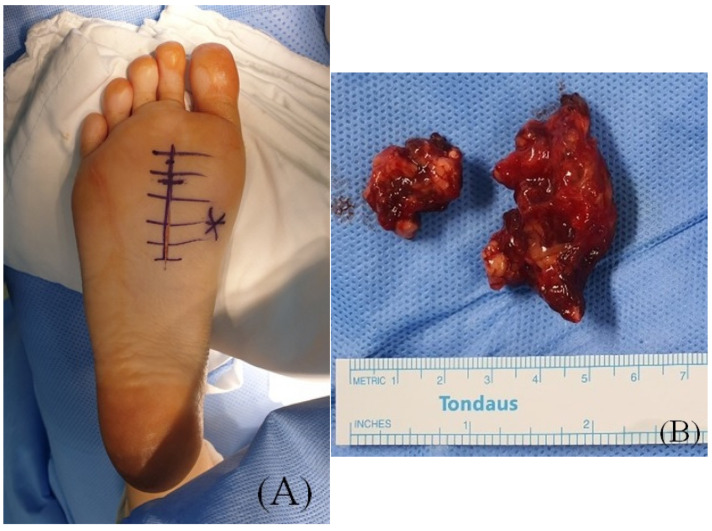
(**A**) Preoperative clinical photographs and incision site. (**B**) Intraoperative photographs of the excised mass.

The patient was prepared in the prone position under spinal anesthesia. A longitudinal incision was made on the second plantar metatarsal shaft (Figure 3A). When we incised skin and dissected the subcutaneous tissue, the bloody multilobulated mass emerged through the plantar fascia correlated with the MRI exam. After dividing the plantar fascia, the mass surrounded by the FDB muscle was removed by wide excision. The mass was soft and irregularly marginated (Figure 3B). 

### 2.3. Postoperative Progression

Postoperatively, the foot and ankle were immobilized for two weeks. The patient was allowed to perform light partial weight-bearing exercise within two weeks. After two weeks, full weight-bearing exercise was permitted. 

**Figure 4:**
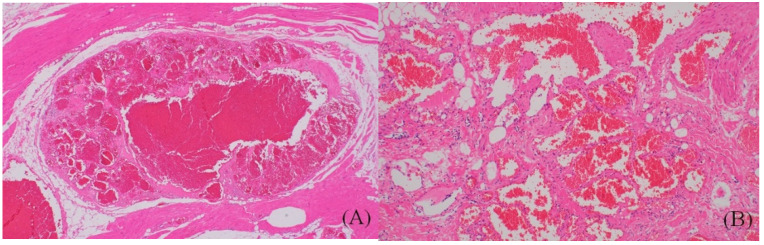
(**A**) Upon low-power examination, dilated vascular structures were noted in the skeletal muscle and fat tissue (hematoxylin and eosin stain, ×40). (**B**) The cavernous-like vascular spaces had variable sized walls and were filled with blood (hematoxylin and eosin stain, ×200).

The patient reported no intermittent pain in the plantar midfoot after surgery. Histologic examination of the excised mass revealed cavernous-like vascular structures within skeletal muscle and adipose tissue, suggesting IH (Figure 4). At the three-month follow-up, the patient was largely asymptomatic and had recovered almost full functionality. She experienced no discomfort during daily life. At one-year follow-up, she exhibited no complications or recurrent symptoms. The VAS and AOFAS scores had improved to 1 and 100 points, respectively. The patient was satisfied with the surgical outcome. 

## 3. Discussion

IHs are benign vascular neoplasms of the skeletal muscle and the most common type of deep soft tissue tumor [14]. IHs account for less than 1% of all hemangiomas, but are slightly more common in young females before the third decade of life [4,11,15]. Malignant transformation is rare [12]. According to the cases reported to date, IHs can develop in any skeletal muscle, but about half of them are found in the lower extremities, with the thigh being the most common site [2,3,16]. Foot-related IHs are rare, with only a few cases being reported in the literature [2,5,6,7,8,9,10,13]. 

The pathogenesis of IH remains unclear. Several authors have suggested a congenital origin [16,17], whereas others have associated IHs with trauma [12,18]. Allen and Enzinger [11] classified IHs into small-vessel (capillary), large-vessel (cavernous), and mixed types based on the vascular lumen and endothelial walls.

Clinical findings of IHs are often similar to those of other soft tissue masses. A palpable mass is typically seen, and it may be asymptomatic or symptomatic, with symptoms including a progressive increase in mass size, persistent pain, functional disability, swelling, increased local temperature, and discoloration of the overlying skin by dilated veins, among others. Although rare, IH can also cause acute compartment syndrome [5].

Various radiological imaging modalities can be used to diagnose IH [19]. Plain radiographs may reveal phleboliths and calcifications, but they are not lesion-specific [19,20,21]. Focal calcification within the mass is also frequent in other soft tissue tumors located in the foot, such as leiomyomas or giant plantar epidermoid cysts. However, in giant plantar epidermoid cyst, the presence of multiple non-shadowing foci different to typical features of phlebolitis of IH [21] is usually frequent. They may also show abnormalities in soft tissue enhancement associated with bony erosion. Color Doppler sonography can help determine the presence or absence of a vascular structure or calcification in the muscle [22]. MRI is the gold standard for diagnosis and is useful for determining the location of the mass and discriminating among the various types [23]. The MRI findings for IH consist of an intermediate- or high-intensity signal in T1-weighted images, often (but not always) accompanied by an intense signal in T2-weighted images [2,19,20]. Griffin et al. [19] reported that 96% of lesions have a hyperintense signal in T2-weighted images relative to skeletal muscle, which is related to dilated vascular spaces filled with stagnant blood. Wu et al. [22] reported that the diagnostic rate of IH using MRI was 90%.

There is still debate over the best treatment for these vascular tumors. Intramuscular sclerotherapy, a kind of radiotherapy, is one well-known option. Uslu et al. [8] reported that sclerotherapy can reduce the size and symptoms of an IH irrespective of its prior size, location, and stage. According to Picci et al. [24] the embolization of peripheral vascular malformations could avoid the need for major intervention. It is easier to treat hemangioma following embolization because this can reduce the operation time and likelihood of bleeding complications. In addition, embolization can be effective for extensively invaded infiltrating hemangioma where complete surgical excision is not possible.

Surgical intervention is the treatment of choice for IH that allows a histopathologic study and reduces recurrence. However, incomplete resection carries a risk of recurrence (the rate of which varies widely; 18–61%) [8,11,12,25]. The mixed type has the greatest tendency for local recurrence (28%), followed by the capillary (20%) and cavernous (9%) types [11].

Because IH in the plantar area is so rare, there is currently no definitive treatment of choice. More study is needed on the pathogenesis, risk factors, recurrence rate, and complications of IH in the plantar area.

## 4. Conclusions

We describe a case of IH in the plantar area. We extracted the tumor through surgery, and the patient had recovered almost completely within a few weeks of the operation. There is debate regarding the treatment of plantar hemangioma. When a patient is not a suitable candidate for surgery, sclerotherapy or combination treatment could also be considered.

## Data Availability

Data sharing is not applicable to this article as no datasets were generated or analysed during the current study.

## Abbreviations

IH = intramuscular hemangioma, VAS = visual analog scale, MRI = magnetic resonance imaging, FDB = flexor digitorum brevis.
